# Electric Field-Driven Water Dipoles: Nanoscale Architecture of Electroporation

**DOI:** 10.1371/journal.pone.0061111

**Published:** 2013-04-11

**Authors:** Mayya Tokman, Jane HyoJin Lee, Zachary A. Levine, Ming-Chak Ho, Michael E. Colvin, P. Thomas Vernier

**Affiliations:** 1 School of Natural Sciences, University of California Merced, Merced, California, United States of America; 2 Department of Physics and Astronomy, University of Southern California, Los Angeles, California, United States of America; 3 Ming Hsieh Department of Electrical Engineering, University of Southern California, Los Angeles, California, United States of America; Universidad de Granada, Spain

## Abstract

Electroporation is the formation of permeabilizing structures in the cell membrane under the influence of an externally imposed electric field. The resulting increased permeability of the membrane enables a wide range of biological applications, including the delivery of normally excluded substances into cells. While electroporation is used extensively in biology, biotechnology, and medicine, its molecular mechanism is not well understood. This lack of knowledge limits the ability to control and fine-tune the process. In this article we propose a novel molecular mechanism for the electroporation of a lipid bilayer based on energetics analysis. Using molecular dynamics simulations we demonstrate that pore formation is driven by the reorganization of the interfacial water molecules. Our energetics analysis and comparisons of simulations with and without the lipid bilayer show that the process of poration is driven by field-induced reorganization of water dipoles at the water-lipid or water-vacuum interfaces into more energetically favorable configurations, with their molecular dipoles oriented in the external field. Although the contributing role of water in electroporation has been noted previously, here we propose that interfacial water molecules are the main players in the process, its initiators and drivers. The role of the lipid layer, to a first-order approximation, is then reduced to a relatively passive barrier. This new view of electroporation simplifies the study of the problem, and opens up new opportunities in both theoretical modeling of the process and experimental research to better control or to use it in new, innovative ways.

## Introduction

Electroporation, also known as electropermeabilization, is the breaching of the integrity of the cell membrane that follows the application of an external electric field of sufficient magnitude and duration. In this process permeabilizing structures (pores) appear in the membrane, allowing molecular transport across this normally impermeable barrier [1]–[3]. Electroporation has a broad range of applications in biology, biotechnology, and medicine, from drug and gene delivery into cells to tumor therapy [4]–[8]. Despite the wide laboratory use of electroporation, the details of the effects of electric fields on biological membranes, and particularly the molecular mechanisms of pore creation in living cells, are not well understood [9]. Our limited knowledge of this phenomenon causes difficulties in controlling the process in clinical applications and limits development of new technologies.

Permeabilization can be monitored by tracking the transport of normally impermeant materials across the cell membrane or by measuring changes in the electrical properties of the membrane, but direct experimental observation is difficult because of the small spatial and fast temporal scales of this process. Theoretical models have been developed to facilitate the interpretation of experimental data and the understanding of the mechanism of electroporation. Although continuum models [10], [11] can predict large-scale features of electroporation, they lack details regarding pore initiation, growth, and decay and contain empirically fitted parameters. Molecular dynamics (MD) simulations provide access to the microscopic structure of a membrane and its interaction with the surrounding solvent and ions in atomic detail. Since we are interested in understanding the molecular mechanism of electropore creation and evolution, we employ MD to study this problem.

Our model focuses on the basic building block of a cell membrane–the phospholipid bilayer. While there is evidence that electroporation is to some extent affected by the complex structure of the cell membrane (e.g. oxidized lipids, cholesterol, lipid heterogeneity, cytoskeletal attachments, etc), the electrical breakdown and the subsequent increase in membrane conductance has long been observed experimentally in simple planar lipid bilayers [12]–[18]. In addition, experimental observations of electroporation due to external nanosecond electric pulses [19] further indicate that while ion channels, cytoskeletal networks, and membrane-associated polypeptides can facilitate additional permeabilization of the membrane, these effects would occur in conjunction with and possibly on a longer time scale than the electrical breakdown of lipid bilayers.

Some MD studies of electroporation have emphasized how phospholipids respond to external electric fields [20]–[22]. The behavior of water dipoles in the complex electric field landscape of the membrane interface has also been noted as an important part of the pore formation process [23]. But the precise cause of pore formation has not been identified, and the roles of phospholipid and water molecules have not been clearly understood. In this article we argue that the electric field-driven reorganization of water dipoles is the primary contributor to electropore formation. We explain pore creation as the result of a rearrangement of interfacial water dipoles into a lower energy configuration in the presence of an external electric field. A scenario for pore formation is presented in which the coherent behavior of water dipoles plays the primary role, while lipids act as somewhat resistive partners in this process. This view simplifies the study of electroporation, stresses the importance of understanding the dynamics of water under the influence of an external electric field in interfacial processes, and opens up new ways to directly connect this problem to those of classical statistical mechanics.

In this work we emphasize the fundamental biophysical interactions between electric fields and molecules in a simple, homogeneous, phospholipid bilayer interface. Although biological membranes are considerably more complex, and electroporation protocols often involve the application of electric fields for much longer times than those considered here, it is our expectation that the model presented will establish the primary events and actors even for more complex descriptions of a membrane.

## Models and Methods

### Model Systems

In order to clearly illustrate the primary role of water dipoles in electropore formation we study two configurations using MD: water-vacuum-water (WVW) and water-lipid-water (WLW).

The WLW systems contain 128 1-palmitoyl-2-oleoyl-*sn*-glycero-3-phosphocholine (POPC) lipids and 4480 water molecules (35 waters/lipid), which results in a system box size of approximately 6.4 nm×6.4 nm×7.2 nm. The two directions tangential to the POPC bilayer are defined as the *X* and *Y* directions, with *Z* perpendicular to the plane of the membrane. To ensure that replicated simulations are independent, each atom was assigned a randomized velocity from a Maxwell distribution at the beginning of a simulation. POPC systems were equilibrated before the application of an external electric field by allowing the simulations to proceed until a constant area per lipid (approximately 0.66 nm^2^) was reached (typically in 10–30 ns).

WVW systems were comprised of 6877 water molecules arranged in two layers of thickness 4.2 nm separated by a 2.8 nm vacuum gap. This configuration was constructed by generating a 7 nm×7 nm×7 nm periodic water box with the GROMACS utility ‘genbox’, then using custom Perl scripts to remove a 2.8 nm slice of water molecules from the center of the system, followed by 300 ps of equilibration at constant volume. The dimensions of this box and the gap size have been chosen carefully in order to produce a realistic equilibrated initial system in which the surface area of the water-vacuum interface is minimized and to ensure that the magnitude of the electric field in the gap is comparable to that of a WLW simulation (Appendix S1). Under non-periodic conditions, the introduction of a vacuum gap into a constant volume system will lead to formation of a spherical bubble that minimizes the area of the water-vacuum interface. However, for a periodic cube, depending on its dimensions and the width of the vacuum gap, one of three possible configurations (a spherical bubble, a tube, and a slab with a vacuum gap in the middle, see Figure S1 in Appendix S1) can minimize the interface surface area given a constant volume. For a cube with a side edge length *L* and a vacuum gap of height *Z*, the water-vacuum gap-water configuration will have the lowest interface surface area, provided that *_Z_* obeys the relationship 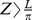 (see Figure S2 in Appendix S1 for details). We verified this theoretical result with MD calculations which confirmed that given a fixed volume of water in the box satisfying the above condition on *Z*, any initial configuration evolves to a minimum surface area state which is indeed comprised of two water layers separated by a vacuum gap. In these validating simulations, the initial system was run for up to 40 ns to ensure that the final state was in fact the equilibrium minimum-surface area configuration. This method allowed us to generate a WVW slab that is stable for tens of nanoseconds, a time scale which is much longer than the characteristic poration time. While the stability of the WVW slab configuration is an artifact of the periodic boundaries, the initial steps in pore formation described in this paper involve a very small volume of water compared to the size of the simulation box and are not affected by the periodicity of the configuration.

After equilibration of the initial WVW and WLW systems we impose an electric field in *Z* direction perpendicular to the membrane surface, and observe the dynamics of the water in the presence of a constant field. We have performed simulations with external electric field values between 450 MV/m and 1000 MV/m and vacuum gap width ranging from 2.8 nm to 4.0 nm (see Table S1) and found no qualitative changes in the system dynamics. Quantitative differences such as reduction in the time scale of the process as the value of the external electric field grows are described in the subsequent sections.

### Molecular Dynamics Simulations Protocols

All simulations were performed using the GROMACS set of programs version 4.5.3, as previously described [24]. The Extended Simple Point Charge (SPC/E) water model [25] was used for all simulations presented here, although we obtained similar results using SPC [26] and SPC/E flexible [27] water models. Lipids are parameterized with OPLS headgroups and Berger hydrocarbon tails [28]. Any atoms which are not explicitly modeled with OPLS or Berger parameters use the native GROMOS87 force field built into GROMACS. Lipid topologies were obtained from Tieleman's group (http://moose.bio.ucalgary.ca).

All simulations were coupled to a temperature bath at 310 K with a relaxation time of 0.1 ps and a pressure bath at 1 bar with a relaxation time of 1 ps, each using a weak coupling algorithm [29]. For lipid systems, pressure was coupled semi-isotropically (using a compressibility of 4.5×10^−5^ bar^−1^) normal to and in the plane of the membrane (NPT). No pressure coupling was used for water-vacuum systems where volume was held constant (NVT). WLW systems were simulated in the NPT ensemble at 1 bar to maintain a dynamically controlled area per lipid before pore formation. WVW systems were run in the NVT ensemble to constrain the box dimensions and water slab separation. Note that despite the different ensemble used for the WLW system, over the time interval of interest the size of the computational box did not fluctuate by more than 0.1 nm without external electric field and 0.5 nm in the presence of electric field.

All simulations were performed with a time step of 2 fs and with Bussi's stochastic velocity rescaling algorithm [30] as a temperature coupling method. Bond lengths were constrained using the LINCS algorithm [31] for lipids and for water. All bond lengths were fixed using constraints after the integration of forces. Following Essmann, et al. [32], the Particle-Mesh-Ewald (PME) method with tinfoil boundary conditions was used to handle long-range electrostatic forces and cut-offs were employed for calculating van der Waal's interactions. In the simulations presented in the next section all of the relevant cut-off distances were set to 1.4 nm. However, we performed simulations with electrostatic cutoffs set to 1.0 nm, 1.2 nm, 1.4 nm, 1.6 nm, and 1.8 nm and found that the results were robust with respect to this parameter and that the dynamics of the pore initiation were qualitatively unchanged.

For systems with an external electric field, the orientation of polar molecules, such as water, in the field induces a net dipole on the system, which has been reported to cause spurious dipole orientations [33]. For the WVW system we investigated several alternative treatments of the long-range electrostatic forces, including PME with non-conducting infinite boundaries, very long cut-offs (3.4 nm), and the reaction field approach, and found that the process of pore initiation is similar, albeit at slightly different electric field strengths. After the pore forms and begins to occupy a significant portion of the computational box, the average dipole saturation differs for different choices of long-range electrostatics approximations and affects the growth rate and stability of the pore. These post-initiation stages in pore development require different models and will be addressed in subsequent publications; here we focus on the pore initiation process.

## Results and Discussion

We concentrate here on the analysis of 60 specific replicate simulations of WLW systems and WVW systems with an external electric field strength of 600 MV/m, but similar results were obtained in additional simulations (hundreds in total) with different thermodynamic ensembles, water models, electrostatic cutoff distances, and applied electric fields, as described above (Table S1). The dynamics of the pore initiation process and the substance of our conclusions regarding its cause are robust and apply in general to all of these cases.

### Dynamics of the Systems

**Figure 1:**
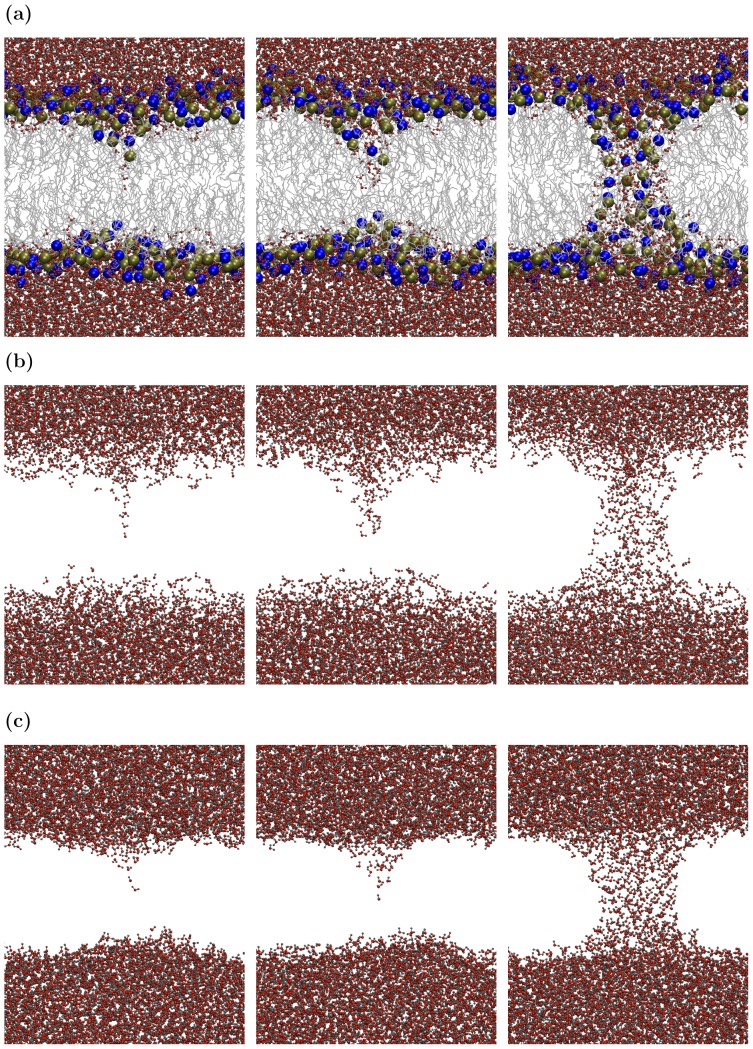
Comparisons of WLW and WVW systems. Snapshots of the time evolution of water-lipid-water (WLW) and water-vacuum-water (WVW) configurations under an external electric field of 500 MV/m. (a) WLW configuration at times 5.8, 6.7, and 7.3 ns from the start of the simulation with both water molecules (oxygen–red, hydrogen-gray) and lipid molecules (phosphorus-yellow, nitrogen-blue, lipid tail groups–silver) displayed. (b) same WLW data as in (a) but with only water molecules shown. (c) WVW configuration at times 1.157, 1.160, and 1.194 ns.

The dynamics described in this subsection were observed in all simulations; we present in detail the results of one representative simulation to illustrate characteristic behavior. Figures 1(a) and 1(b) show the time evolution of a water-phospholipid bilayer-water (WLW) configuration over 14 ns. Fig. 1(a) and 1(b) render the same data set, but Figure 1(a) displays both water and lipid molecules, while Figure 1(b) shows only water molecules. Pore formation can be roughly described as a three-part process. First, a deformation directed towards the interior of the membrane forms at the water-lipid interface. Second, this bump grows, and eventually water molecules from one side of the bilayer meet water from the opposite side to form a bridge, closely followed by lipid head groups. Third, the newly formed column of water between the two sides of the bilayer expands to become a lipid pore–a structure with water molecules in the middle and lipid head groups lining the periphery. A detailed description of this process can be found in earlier publications [20], [23], [24].

Figure 1(b) shows the process of pore formation from the perspective of water dynamics. Pore formation starts with protrusions consisting of a few water molecules, often as a single-file column, appearing either side of the bilayer. A protrusion extends into the bilayer interior and then expands at the base, forming a conical structure which eventually bridges the membrane. The radius of the water column spanning the bilayer then begins to increase.

Now let us examine the water-vacuum-water (WVW) simulations. Figure 1(c) displays the time evolution of the molecules in the water slabs under the influence of an externally imposed electric field. The progression of the dynamics of water column formation is very similar to the behavior of water in the WLW system (compare Figures 1(b) and 1(c)). The dynamics of pore (water column) formation and the similarity between WLW and WVW simulations are invariant across simulations over a wide range of parameters (e.g. external electric field between 450 MV/m and 1000 MV/m, vacuum gap width ranging from 2.6 nm to 4.0 nm).

**Figure 2:**
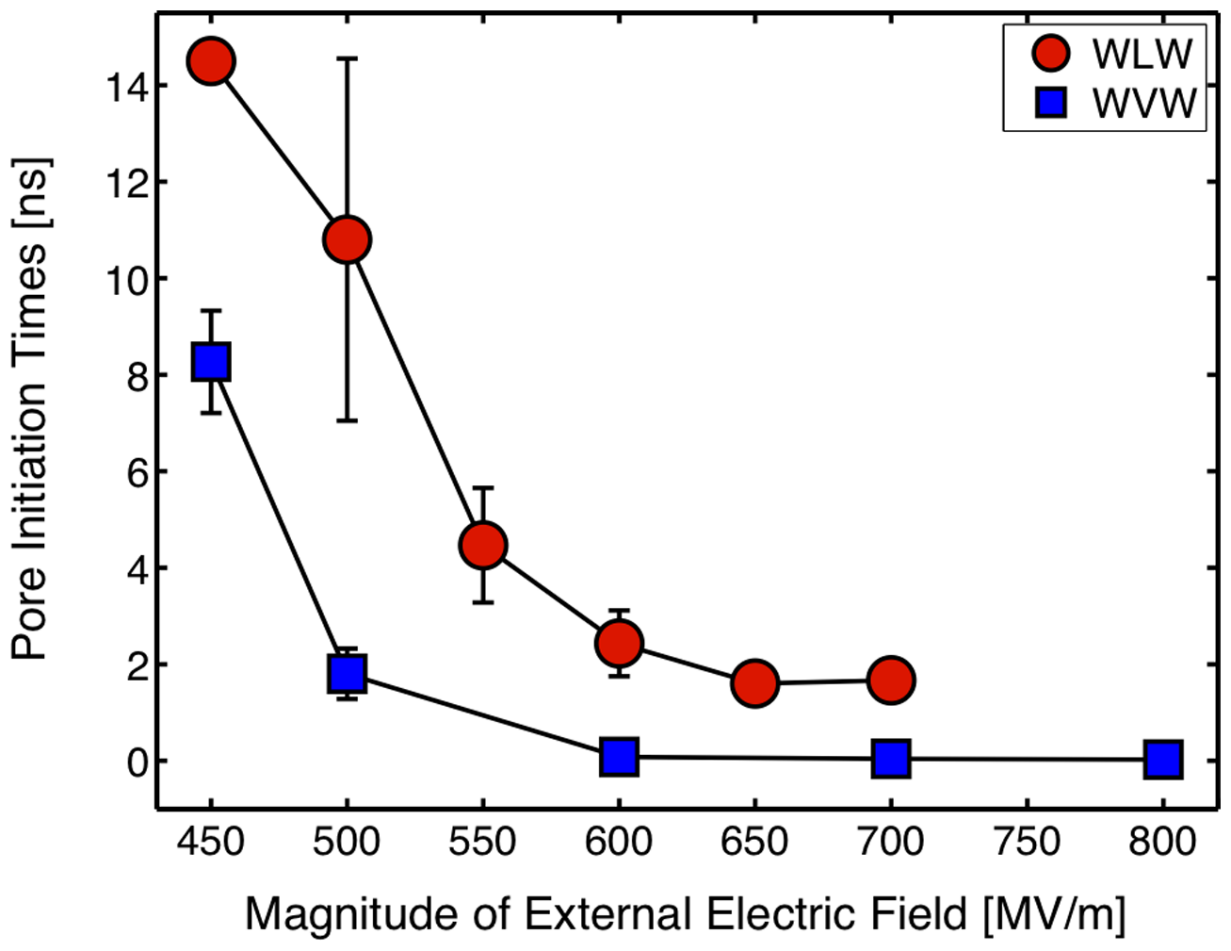
Pore initiation times. Average pore initiation times for WLW and WVW systems calculated with three sets of simulations for each configuration.

WLW and WVW simulations differ mainly in the time scale over which the formation of the water bridge occurs, i.e., the pore initiation time [24]. To compare initiation times between WLW and WVW systems, we selected a gap size for WVW systems that result in the same magnitudes of the external and internal (in the lipid bilayer interior and the vacuum gap) electric fields. This ensures that the interfacial water molecules are exposed to similar electric fields in the WLW and WVW systems, permitting a fair comparison of pore initiation times (see Appendix S1). With equivalent external and internal electric fields, WVW systems porate faster than WLW systems (Fig. 2). We hypothesize that the lipid bilayer acts as a barrier to the interfacial water dipoles, retarding the formation of the water bridge connecting the two water layers. The energetics analysis presented in the next sections supports this hypothesis.

### Energetics Analysis of the Systems

We now discuss the energetics of the simulated systems. We demonstrate that the pore formation process described above is driven by the collective tendency of the interfacial water dipoles to minimize their electrostatic interactions while adopting an orientation that minimizes the energy of the water dipole in the external electric field, reflected in the steady drop in the per-molecule energy of waters in the nascent pore as the protrusion develops. Below we demonstrate that this energetic behavior is present in both WVW and WLW simulations.

In order to carry out the analysis, we developed the following tools to examine the energetics of water protrusion formation in ten WLW and ten WVW simulations. First, we define the interface region of the water as follows. The computational box is split in the *Z* dimension into rectangular slices of thickness 0.1 nm. In each slice the average density of water molecules is calculated, and the highest value is defined as bulk density. Slices with water density not exceeding 50% of the bulk value are considered interfacial, and those with water density equal to or greater than 50% of the bulk value are treated as bulk.

In order to compute the time history of the protrusion energetics, we must identify the water molecules directly involved in protrusion formation. Note that the trajectory of an individual water molecule is very noisy so that selecting protrusion molecules for each time frame is both impractical and arbitrary. Instead we select a rectangular box that includes all of the water molecules we consider to be in the protrusion at the end of our time interval of interest, i.e. at the time when pore initiation is complete, then work backwards to track all water molecules located in this box in each preceding time frame.

To define the box containing water protrusion molecules we carefully inspect the VMD [34] visualizations of the data to find the point in time when the water molecules in the protrusion growing from one of the water layers join with the water molecules from the opposite water layer. At this time point–the end of protrusion initiation time and the beginning of pore formation–we find the coordinates of the extent of the box containing protrusion water molecules in *X* and *Y* and the height of the protrusion, i.e. the coordinate of the top of the protrusion box, in *Z*. The base of the protrusion box in *Z* coincides with the boundary of the interface region (i.e. the point above which the density of water does not exceed 50% of the bulk water density). The coordinates of the protrusion box containing this set of water protrusion molecules are then set for all time frames.

Once we isolate the water molecules comprising the protrusion, we compute the time history of the average potential energy per protrusion molecule and its constituent terms, specifically: (i) the electrostatic interaction energy between water molecules in the protrusion and all other water molecules, (ii) the Lennard-Jones approximation to the van der Waal's interaction between protrusion and bulk water, and (iii) the interaction energy between the protrusion water dipoles and the external electric field (see Appendix S2 for functional forms of the energetic terms). For WLW simulations we also compute the electrostatic and Lennard-Jones interaction energies between the protrusion water molecules and the lipids. Note that all energetic terms are computed as averages per protrusion molecule. That is, at each time frame we determine how many water molecules are in the protrusion box and divide each of the interaction energy values by the number of protrusion molecules. The details of the energy terms calculations can be found in Appendix S2.

In addition to the energy terms, we calculate the height *H* of the protrusion as the distance between the interface region boundary and the protrusion atom (i.e. an atom in the protrusion box), which is the farthest from this boundary in *Z*. Since the data is noisy, for each variable we also compute a smoothed version using a 50 ps moving average for WLW and a 2 ps moving average for WVW simulations.

**Figure 3:**
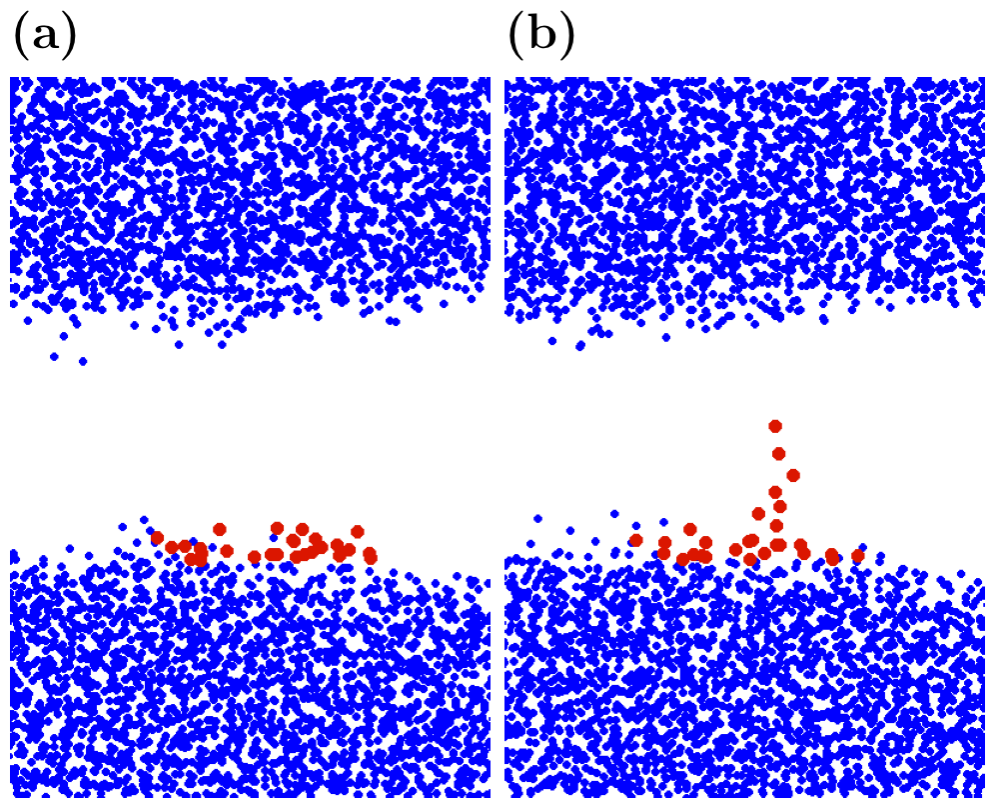
Protrusion molecules identification. XZ-projection of the water molecules positions in a typical WVW simulation at the times (a) just before protrusion begins to grow and (b) just before it begins to interact with/attract water molecules from the other side of the gap. Protrusion molecules are colored in red and the rest of the water molecules are shown as blue.

For each of the simulations we use visualization of data to determine a time interval over which the tip of the protrusion extends from approximately the top of the interface layer to roughly half of the height of the middle region free of water molecules. In this way we capture the protrusion just after it begins to grow and just before it starts interacting with the water molecules in the layer on the other side of the vacuum gap or lipid bilayer. Since there is so much variation between replicate simulations, we determine this time interval for each simulation individually both from the perspective of the protrusion height as well as the energy variables. Figure 3 illustrates typical positions of the protrusion and the rest of the water molecules at the initial and the final points of the identified time interval. The figure displays the data from a single WVW simulation but the rest of the WVW and all of the WLW simulations yield a qualitatively similar picture.

**Figure 4:**
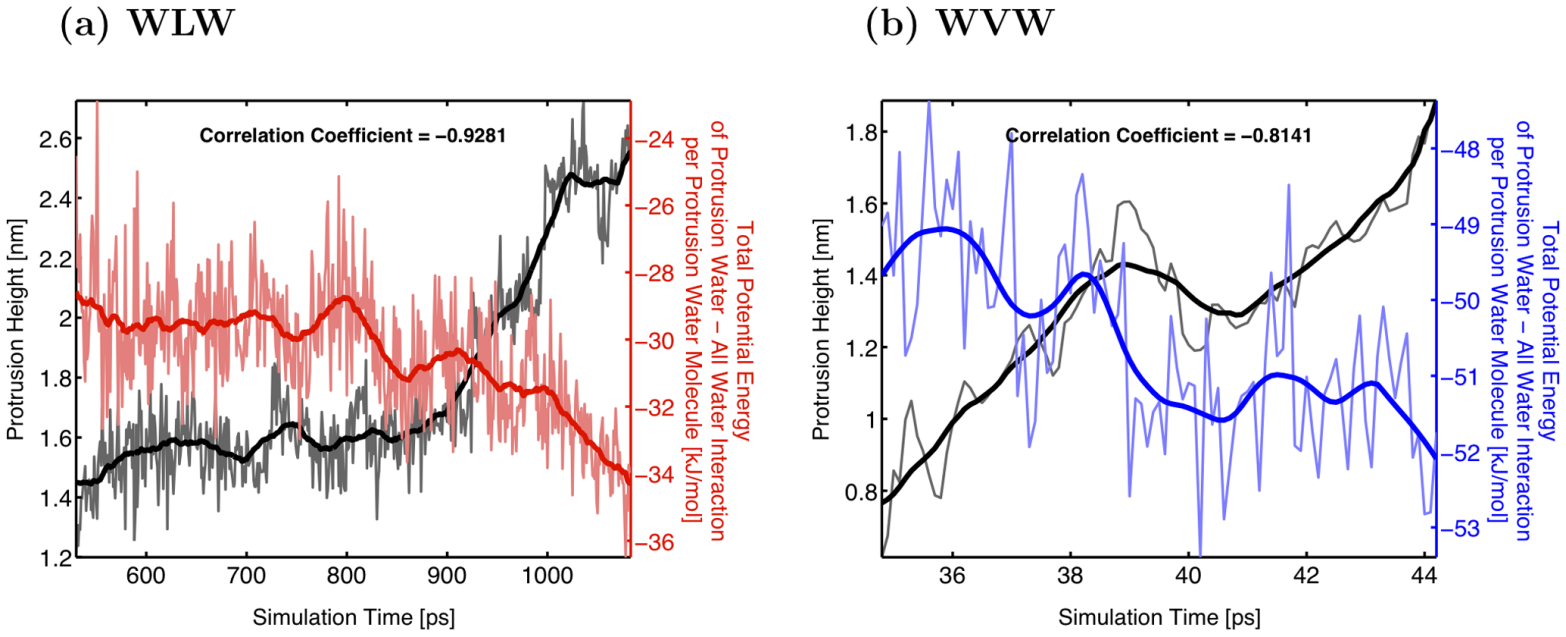
Anti-correlation of protrusion height and total interaction energy. Graphs demonstrating anti-correlation between the increase of the protrusion height (black curve) and the decrease of the total interaction energy per protrusion molecule of protrusion waters with all other water molecules (both in the protrusion and in bulk) for (a) WLW (red curve) and (b) WVW (blue curve) simulations.

This analysis reveals a drop in the per-molecule energy of the waters in the protrusion as the height of the protrusion grows for both WLW and WVW (Fig. 4). To show a correlation between this drop in energy and the protrusion growth for each simulation we computed the Pearson's correlation coefficient between the smoothed data for protrusion height and each of the energies per protrusion water molecule over the time interval described above (mid-point of protrusion growth).

**Figure 5:**
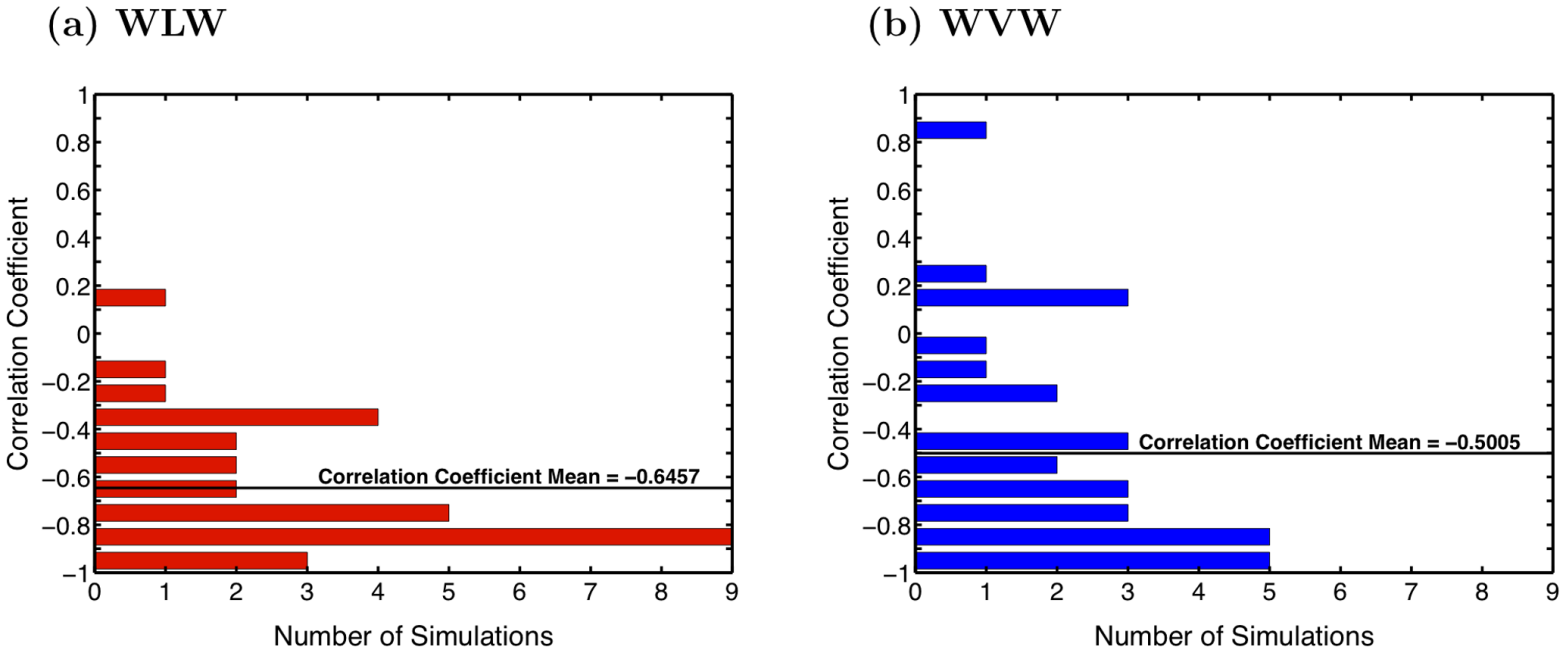
Pearson correlation coefficients. Histograms of the Pearson correlation coefficients demonstrating anti-correlation between the increase of the protrusion height and the decrease of the total interaction energy per protrusion molecule of protrusion waters with all other water molecules (both in the protrusion and in bulk) for (a) WLW and (b) WVW simulations.

First, we examine the correlation coefficient between the protrusion height and the sum of all the energetic interaction terms (i), (ii) and (iii) between the protrusion water and the bulk water. Figure 5 displays histograms of the correlation coefficient distribution across the WLW (Fig. 5a) and WVW (Fig. 5b) simulations. For the thirty WLW simulations this correlation coefficient has a mean value of −0.65 and a median value of −0.76. Note that the correlation coefficient is negative for 29 of the 30 thirty simulations. Under close inspection we find that the one simulation with a positive correlation coefficient exhibits anomalous protrusion formation. In this case some of the water molecules become detached from the bulk for a portion of the pore formation process. This makes it difficult to identify and isolate the protrusion molecules using our procedure. The results for the 30 simulations clearly demonstrate, however, that the growth of the protrusion is coupled with a drop in the average interaction energy for the water molecules in the protrusion.

The results for the WVW simulations are noisier, since water molecules move more freely into and out of the interface and protrusion regions than they do in WLW systems, where the interface water mobility is tempered by the presence of lipid headgroups. The mean of the correlation coefficient is −0.5, and the median is −0.63. Despite the noisier data, for 25 of 30 WVW simulations the correlation coefficient is negative. In four of the five WVW simulations with positive correlation coefficients we find that fluctuations in the extent of the protrusion during its growth make it difficult to isolate the protrusion molecules in a rectangular box. In the fifth simulation (correlation coefficient 0.82) we find two protrusions growing in close proximity, and the interaction between the two protrusions appears to affect the dynamics. Isolating the energetics of one of the protrusions is difficult and incomplete, since it is significantly affected by the neighboring protrusion. The deviation from the negative correlation in these exceptional cases does not alter the overall conclusion for the energetics of protrusion water molecules. Statistically both in WLW and WVW simulations we see a clear correlation between the growth of the protrusion and the decrease of the per protrusion water molecule interaction energy between the protrusion water and the bulk water.

**Figure 6:**
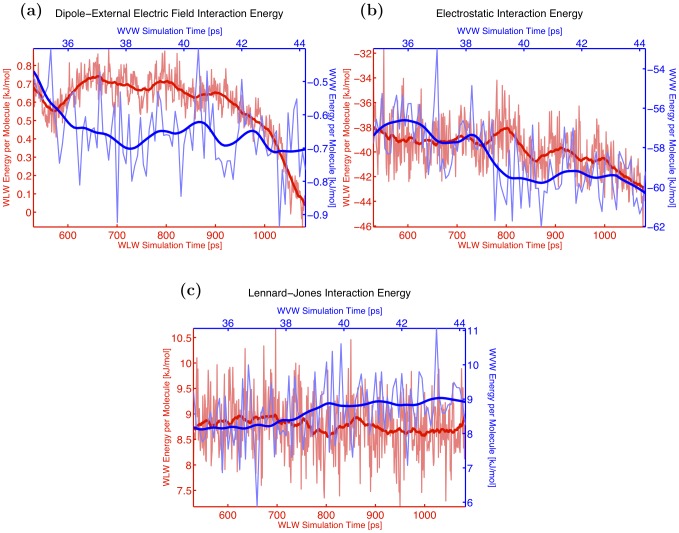
Constituent terms of total interaction energy. Comparison of constituent terms of the total interaction energy per protrusion molecule between WLW (red curve) and WVW (blue curve) simulations: (a) dipole–external electric field interaction energy, (b) electrostatic interaction energy, (c) Lennard-Jones interaction energy.

**Figure 7:**
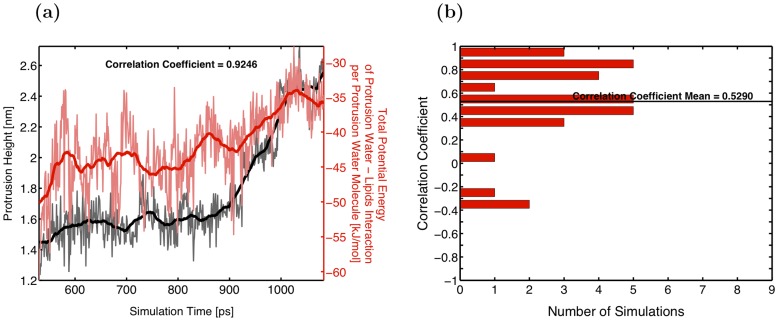
Correlation between protrusion height and total interaction energy in WLW. A graph (a) and a histogram of the correlation coefficient (b) demonstrating positive correlation between the protrusion height growth and the increase in the total interaction energy between the protrusion waters and the lipids in WLW simulations.

In order to illustrate the details of the interaction energies evolution as the protrusion grows we choose two representative simulations, a WLW and a WVW system, and examine their energetics. The results of this comparison are presented in Figures 4, 6 and 7. From Figure 4 we can see why the correlation coefficient in both cases is negative: as the height of the protrusion grows (green curve), the sum of total protrusion-protrusion and protrusion-bulk water interaction energies per protrusion molecule (WLW–red curve, WVW–blue curve) decreases. Note that over the course of the identified time interval the magnitude of the decrease in the total potential energy per protrusion molecule is comparable for all sixty WLW and WVW simulations, with WLW systems showing a slightly higher decrease. On average the protrusion water molecule energy in WLW simulations is reduced by approximately 2.8 kJ/mol, while for the WVW protrusion waters the total potential energy decreases by 1.4 kJ/mol.

In Figure 6 we decompose these total energies into the three components discussed above, (i), (ii) and (iii), to illustrate how this decrease is accounted for. The potential energy decrease is mainly due to the decrease of the electrostatic interaction energy (Fig. 6(a)). The dipole-external electric field interaction term decreases slightly (Fig. 6(b)), and the Lennard-Jones potential energy remains largely unchanged (Fig. 6(c)). The decrease in the dipole–electric field interaction term corresponds with the alignment of the protrusion molecules with the external field. To examine this alignment more closely we also compute an average angle between water dipoles and the electric field in the protrusion and in the bulk. We find that from the start of a simulation to the end of the pore initiation process in the thirty WVW systems this angle is reduced by 19 degrees on average for the protrusion waters and by only 4 degrees in the bulk. Similarly, in the thirty WLW systems the dipole-electric field angle decreases by 11 degrees in the protrusion and only by 2 degrees in the bulk.

To summarize, these results present a scenario of protrusion evolution in which the interfacial waters, constrained less than the molecules in the bulk, align with the external field and are restructured into a protruding column, where the dipoles are arranged vertically in a configuration that is energetically more favorable than a horizontal arrangement in the plane of the membrane. The external electric field drives the water molecules to overcome their interfacial and bulk interactions and to form the protrusion (see Appendix S4 for desolvation energy estimates).

The fluctuations in the WVW system are larger, but the overall energetic behavior of the protrusion molecules is similar between WLW and WVW systems and consistent throughout all thirty simulations. The main difference between WLW and WVW dynamics is the time scale of protrusion and pore formation. In the WVW case the water molecules are more mobile and free to form a protrusion since they are not restrained by the lipid membrane.

The retarding effect of the phospholipids on water protrusion formation can be quantitatively verified by an examination of the protrusion waters–*lipids* interaction energy. For the WLW simulations we have computed a correlation coefficient between the protrusion height and the sum of the electrostatic and Lennard-Jones interaction energies between protrusion water molecules and the lipids in the system. As before, this term is calculated per protrusion water molecule. For the thirty WLW simulations the correlation coefficient ranges from −0.38 to 0.96 with a mean of 0.53 and a median of 0.57. Figure 7 shows a typical evolution as the protrusion–lipid interaction energy increases with the growth of the protrusion height. Note that while Figure 7 displays interaction energy between protrusion waters and all other lipids in the system, we also confirmed that it is primarily the neighboring lipids (i.e. lipid molecules which have at least one headgroup atom located within 5 Angstroms of any protrusion atom) that contribute to this energy. Appendix S3 illustrates this point in detail (e.g. see Figure S3 in Appendix S3). Given the positive correlation between the growth of the protrusion and the protrusion water-lipids interaction energy increase, we conclude that as the protrusion forms, the initial interaction between the protrusion molecules and the lipids is unfavorable and lipids serve as a barrier delaying the formation of the protrusion.

While it is not possible to consider the protrusion molecules as an isolated subsystem with self-contained energetics and clearly decreasing total energy, our analysis reveals similarities between the WVW and WLW systems at both structural and energetic levels. Based on these results we argue that the same mechanism of electrostatic energy minimization in the presence of external electric field drives formation of pores for both WLW and WVW configurations, but the presence of lipid bilayer slows down this process.

**Figure 8:**
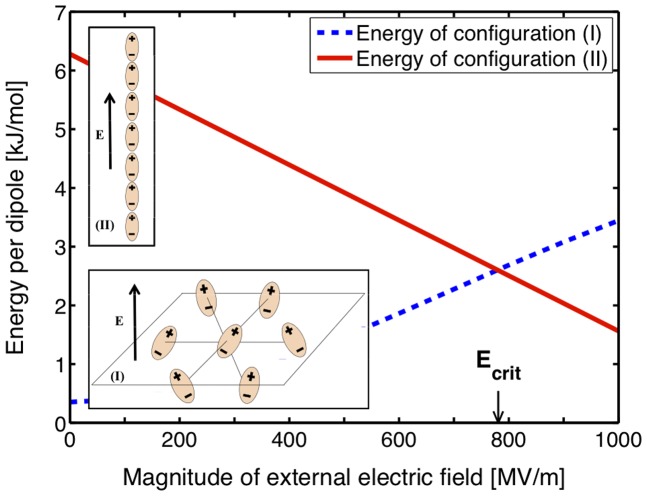
Energetic comparison of vertical vs. planar dipole configurations. Total energies of dipole configurations (I) and (II). Dashed line–sum of dipole-dipole interaction and dipole-electric field interaction terms for a horizontal layer of oriented dipoles (configuration (I)), solid line–sum of dipole-dipole interaction, dipole-electric field interaction, and the total solvation energy required to remove the dipoles from the bulk water for the vertical stack of dipoles (configuration (II)).

The following simple theoretical model illustrates the results of the simulations and the energetic benefits of a protrusion creation. Consider two configurations of 7 dipoles: (I) a horizontal (i.e. perpendicular to the external electric field direction) sheet of equidistant dipoles, where the mean dipole component 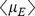 in the direction of the external electric field *_E_* is set according to the theoretically established dependency of 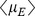 on the magnitude of *E* in bulk water (this can be computed analytically for a system of dipoles from a Langevin-Debye formula [35], 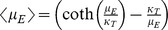, or deduced from MD simulations [22]); (II) a vertical (i.e. parallel to the external electric field) single-file chain of equidistant dipoles aligned with the external electric field. The distance between dipoles is set to 0.31 nm in agreement with the average spacing of water molecules in the SPC/E model. We now compute and compare the average energies of configurations (I) and (II) as follows. The total energy for (I) is comprised of two terms: the dipole-dipole interaction, which represents the dominant term of the electrostatic interaction between all of the dipoles, and the energy of the dipole in the external electric field. In addition to these two terms the total energy for configuration (II) includes an estimate of the desolvation energy it would require to remove the dipoles from the bulk water [36] (see Appendix S4 for details). For each of the two configurations we computed these total energies for different values of an external electric field ranging from 0 to 1000 MV/m. Figure 8 shows the dependence of the total energy of each of the configurations on the external electric field values. The results presented in the figure demonstrate that we can expect the existence of a critical field value 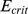 such that creation of configuration (II) becomes more energetically favorable then aligning the dipoles in the bulk water. While obviously we cannot expect this basic model to provide a precise estimate of the 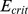 value, it illustrates our theory of formation of protrusions and bridges at the water-vacuum interface as a result of electrostatic energy minimization.

## Conclusions

Our simulations and analysis show that at the molecular scale electroporation of a phospholipid bilayer is driven by the restructuring of interfacial water molecules into column-like structures as their dipole moments align with an external electric field. This electric field-driven reorganization of the lipid bilayer is associated with an overall drop in the per-molecule energy for the waters in the growing protrusion. Membrane phospholipids simply follow the bridging water. This view allows significant reduction of the complexity of the analysis of electroporation and opens possibilities for applying well-developed analytical and computational tools to study this problem. Additionally, this insight into the significance of the interfacial water dynamics can facilitate development of new experimental and technological approaches to better control and utilize the process of electroporation.

## Supporting Information

Table S1 **List of the performed simulations and the associated parameters.**
(XLSX)

Appendix S1 **Choosing initial water-vacuum-water (WVW) configuration.**
(DOCX)

Appendix S2 **Computing interaction energies for WVW and WLW configurations.**
(DOCX)

Appendix S3 **Details of the protrusion waters–lipids interaction energy calculations.**
(DOCX)

Appendix S4 **Computing energetic interactions of planar and vertical configurations of seven dipoles.**
(DOCX)
