# Supplementary material for: Electric Field-Driven Water Dipoles: Nanoscale Architecture of Electroporation
Source: PLoS One. 2013 Apr 11;8(4):e61111. doi: 10.1371/journal.pone.0061111 (PMC3623848; doi:10.1371/journal.pone.0061111)
Supplement: Appendix S1 — Choosing initial water-vacuum-water (WVW) configuration. (DOCX) [file pone.0061111.s002.docx]

**SUPPORTING INFORMATION: Appendix S1**

**Title:** Electric Field-Driven Water Dipoles: Nanoscale Architecture of Electroporation

**Authors and Affiliations:** Mayya Tokman^†^, Jane HyoJin Lee^†^, Zachary A. Levine^‡^, Ming-Chak Ho^‡^, Michael E. Colvin^†^, P. Thomas Vernier^§^

*^†^School of Natural Sciences, University of California, Merced, CA, USA.*

*^‡^Department of Physics and Astronomy, University of Southern California, Los Angeles, CA, USA.*

*^§^Ming Hsieh Department of Electrical Engineering, University of Southern California,* *Los Angeles, CA, USA.*

**Corresponding Author:** Mayya Tokman, School of Natural Sciences, University of California, 5200 N. Lake Road, Merced, CA 95343, [mtokman@ucmerced.edu](mailto:mtokman@ucmerced.edu).

**APPENDIX S1: Choosing initial water-vacuum-water (WVW) configuration.** A stable initial configuration has to be chosen to perform reliable simulations of WVW systems. In the absence of the external electric field we expect the water molecules to be arranged so that the surface area of the water-vacuum interface is minimized. Such configuration will then be stable and can be used as the initial condition for WVW simulations. To find such configuration let us consider a cubic box with side lengths $L$, containing a fixed volume of water $V$ (Fig. S1a in Appendix S1). It is easy to see that there are three possible candidate configurations that can have a minimum surface area of the water-vacuum interface: (i) a rectangular vacuum layer of width $Z$, extending to the boundary and separating two rectangular water layers, i.e. a “sandwich” (Fig. S1b in Appendix S1), (ii) a cylindrical vacuum tube extending to the boundary and surrounded by water (Fig. S1c), and (iii) a spherical vacuum bubble surrounded by water (Fig. S1d in Appendix S1). Since the volume is fixed for all three configurations, we can replace the parameter $V$ with a parameter $Z$ that corresponds to the vacuum layer width for the “sandwich” configuration. We can then calculate the surface area for all three configurations in terms of the parameters $Z$ and $L$ (see formulas in Fig. S1 in Appendix S1). Thus for a given value of $L$ we can compute the surface area $A$ for a range of $Z$ values and determine for each value of the $Z$ a configuration with the smallest surface area. Simple algebraic manipulations of the formulas for the areas of the three configurations lead to the condition that the “sandwich” configuration will have the lowest surface area if $Z$ obeys the relationship $Z>\frac{L}{\pi}$. Figure S2 in Appendix S1 shows corresponding graphs for $L=7$ nm; as we can see, for $Z=2.8$ nm the “sandwich”, or the vacuum slab, configuration has the lowest surface area.

Now we need to choose the appropriate value for$Z$ which satisfies $Z>\frac{L}{\pi}$. To ensure fair comparison with the water-lipid-water systems we need to choose the size of the gap so that the magnitude of the electric field experienced by the water molecules at the interface is comparable between the WVW and the WLW systems. It is not appropriate to compare the externally applied electric fields for the two systems since the presence of lipids will influence the magnitude of the electric field at the interface. On the other hand the size of the vacuum gap will have an effect on the resulting electric field at the water-vacuum interface for the WVW configuration. For the WLW system the membrane size is set by the mean separation of the POPC glycerol acyl oxygen atoms from one lipid leaflet to the other. Thus to choose the gap size for the WVW system we first apply different values of the external electric field to the WLW system and measure the resulting internal electric field at the water-lipids interface. We then vary the vacuum gap size $Z$ in the WVW configuration for the same range of the external electric field magnitudes to obtain the same values of the internal electric field at the water-vacuum interface as in the water-lipid interface case. Such approach led us to choose the value of $Z$ to be 2.8 nm. For this value of $Z$ both the external and the internal interface electric fields magnitudes agree between the WLW and the WVW systems, thus the comparison of poration times (Fig 2) is fair.

**Figure S1.** *Three possible minimum surface area configurations for a cubic box containing fixed volume of water: (a) “sandwich”, (b) tube, and (c) spherical bubble.*

**
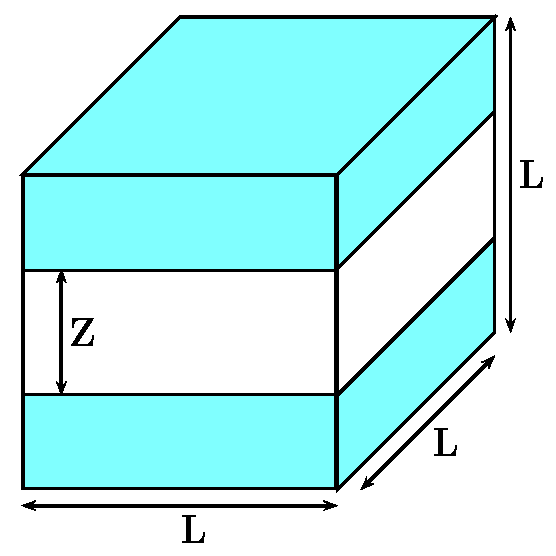

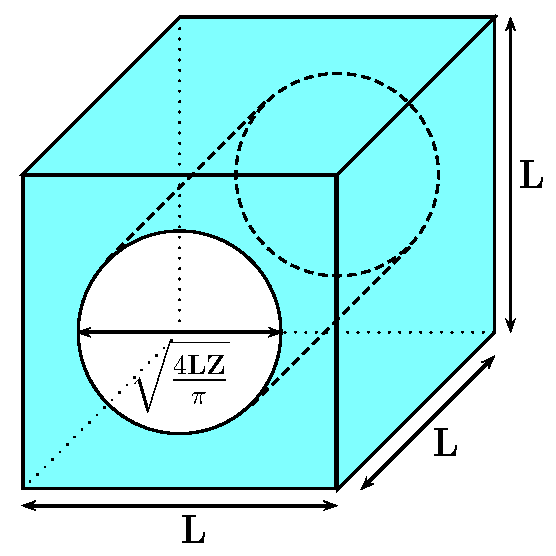

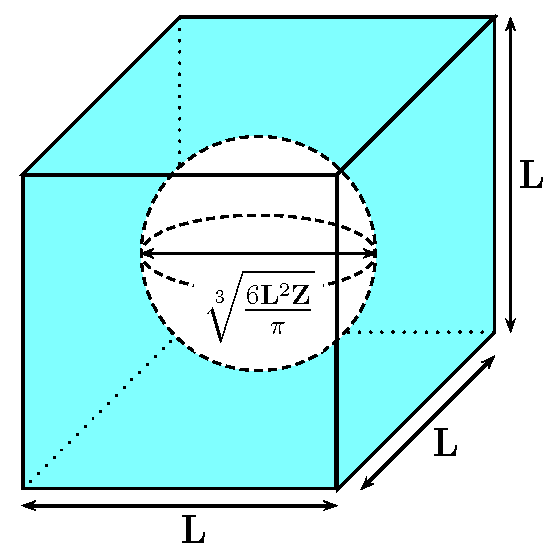
**

(a) ${A =2L}^{2}$ (b) $A =2\sqrt{\pi L^{3}Z}$ (c) $A =4\pi\left( \frac{3L^{2}Z}{4\pi} \right)^{\frac{2}{3}}$

**Figure S2.** *Surface area of the three potential minimum area configurations for a fixed value of L=7nm and a varying value of Z. For Z = 2.8nm the “sandwich” configuration has the lowest surface area.***
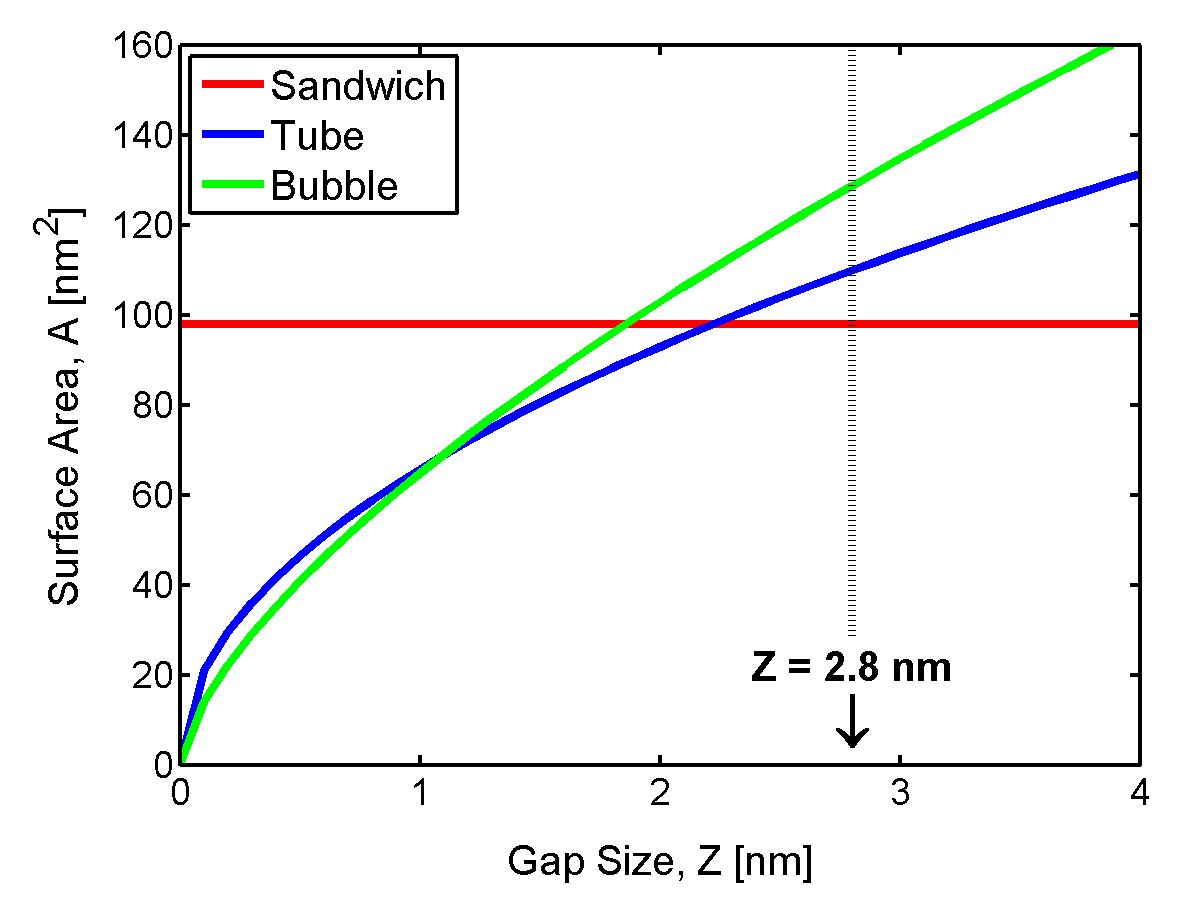
**
