# Supplementary material for: Electric Field-Driven Water Dipoles: Nanoscale Architecture of Electroporation
Source: PLoS One. 2013 Apr 11;8(4):e61111. doi: 10.1371/journal.pone.0061111 (PMC3623848; doi:10.1371/journal.pone.0061111)
Supplement: Appendix S2 — Computing interaction energies for WVW and WLW configurations. (DOCX) [file pone.0061111.s003.docx]

**SUPPORTING INFORMATION: Appendix S2**

**Title:** Electric Field-Driven Water Dipoles: Nanoscale Architecture of Electroporation

**Authors and Affiliations:** Mayya Tokman^†^, Jane HyoJin Lee^†^, Zachary A. Levine^‡^, Ming-Chak Ho^‡^, Michael E. Colvin^†^, P. Thomas Vernier^§^

*^†^School of Natural Sciences, University of California, Merced, CA, USA.*

*^‡^Department of Physics and Astronomy, University of Southern California, Los Angeles, CA, USA.*

*^§^Ming Hsieh Department of Electrical Engineering, University of Southern California,* *Los Angeles, CA, USA.*

**Corresponding Author:** Mayya Tokman, School of Natural Sciences, University of California, 5200 N. Lake Road, Merced, CA 95343, [mtokman@ucmerced.edu](mailto:mtokman@ucmerced.edu).

**APPENDIX S2: Computing interaction energies for WVW and WLW configurations.** The analysis presented in the manuscript involves computing interaction energies between various parts of the system. Specifically, we calculate the electrostatic, the Lennard-Jones and the dipole-external electric field interaction energies. The following formulas are used to compute respective interaction. For each two atoms denoted i and j that comprise either a water or a lipid molecule the electrostatic (Coulomb) interaction energy is given by

$$U_{E}\left( r_{\mathrm{ij}} \right)=\frac{1}{4\pi\varepsilon_{0}}\frac{q_{i}q_{j}}{\left\| r_{\mathrm{ij}} \right\|},$$

(S1)

where $\varepsilon_{0}$ is the vacuum permittivity, $q_{i}$, $q_{j}$ are the atoms’ charges and $\left\| r_{\mathrm{ij}} \right\|$ is the distance between the charges, i and j. To calculate Van der Waal’s interaction between these atoms we use the Lennard-Jones approximation to the interaction potential and compute

$$U_{\mathrm{LJ}}\left( r_{\mathrm{ij}} \right)=4\varepsilon_{\mathrm{ij}}\left[ \left( \frac{\sigma_{\mathrm{ij}}}{\left\| r_{\mathrm{ij}} \right\|} \right)^{12}-\left( \frac{\sigma_{\mathrm{ij}}}{\left\| r_{\mathrm{ij}} \right\|} \right)^{6} \right],$$

(S2)

where ${\varepsilon_{\mathrm{ij}}=\left( \varepsilon_{i}\varepsilon_{j} \right)}^{1/2}$ , $\varepsilon_{i}$ $,\varepsilon_{j}$ are the depths of the atoms’ potential wells and $\sigma_{\mathrm{ij}}=\frac{1}{2}\left( \sigma_{i}+\sigma_{j} \right)$ is the average of the Van der Waal’s radii of the atoms. Finally, we also compute the interaction energy between water dipoles and the external electric field $E$ as

(S3)

$$U_{\mu}=E\mu\left( 1-\cos\theta\right).$$

Here $\mu$ is the dipole moment of a water molecule and$\theta$ is the angle between the electric field and the dipole moment. In the main text we consider either individual energy terms specified by equations (S1, S2, S3) in Appendix S2 or their total, summed over all pairwise interactions between the atoms of the regions of interest. When we consider interaction energy among the protrusion waters and between the protrusion and bulk molecules, we add all the pairwise interactions accordingly and compute the average energy per protrusion molecule. We calculate protrusion – lipids interaction in a similar way.
