# Supplementary material for: Electric Field-Driven Water Dipoles: Nanoscale Architecture of Electroporation
Source: PLoS One. 2013 Apr 11;8(4):e61111. doi: 10.1371/journal.pone.0061111 (PMC3623848; doi:10.1371/journal.pone.0061111)
Supplement: Appendix S3 — Details of the protrusion waters–lipids interaction energy calculations. (DOCX) [file pone.0061111.s004.docx]

**SUPPORTING INFORMATION: Appendix S3**

**Title:** Electric Field-Driven Water Dipoles: Nanoscale Architecture of Electroporation

**Authors and Affiliations:** Mayya Tokman^†^, Jane HyoJin Lee^†^, Zachary A. Levine^‡^, Ming-Chak Ho^‡^, Michael E. Colvin^†^, P. Thomas Vernier^§^

*^†^School of Natural Sciences, University of California, Merced, CA, USA.*

*^‡^Department of Physics and Astronomy, University of Southern California, Los Angeles, CA, USA.*

*^§^Ming Hsieh Department of Electrical Engineering, University of Southern California,* *Los Angeles, CA, USA.*

**Corresponding Author:** Mayya Tokman, School of Natural Sciences, University of California, 5200 N. Lake Road, Merced, CA 95343, [mtokman@ucmerced.edu](mailto:mtokman@ucmerced.edu).

**APPENDIX S3: Details of the protrusion waters – lipids interaction energy calculations.**  While Figure 6 of the main text displays per protrusion molecule interaction energy between all lipids in the system and protrusion waters, we also calculated this energy including only lipids neighboring the protrusion as follows. We have found all lipid molecules which have at least one headgroup atom located within 5 Angstroms of any protrusion atom. We designated these lipids as neighboring the protrusion and computed two interaction energies - between protrusion waters and the neighboring lipids only (Figure S3(a) in Appendix S3) and between protrusion waters and the rest of the lipids (Figure S3(b) in Appendix S3). As Figure S3 in Appendix S3 and Figure 7 demonstrate that the neighboring lipids only make any significant contribution to the protrusion-lipids interaction energy.

**Figure S3.** *Per-protrusion-molecule interaction energy between protrusion and (a) only the neighboring lipids and (b) the rest of the lipids, excluding neighboring lipids. By comparing these graphs with Figure 7, which displays the sum of the terms in Fig S3(a) and S3(b), note that the primary contribution to the protrusion-lipids interaction energy comes only from the lipids neighboring the protrusion.*

*
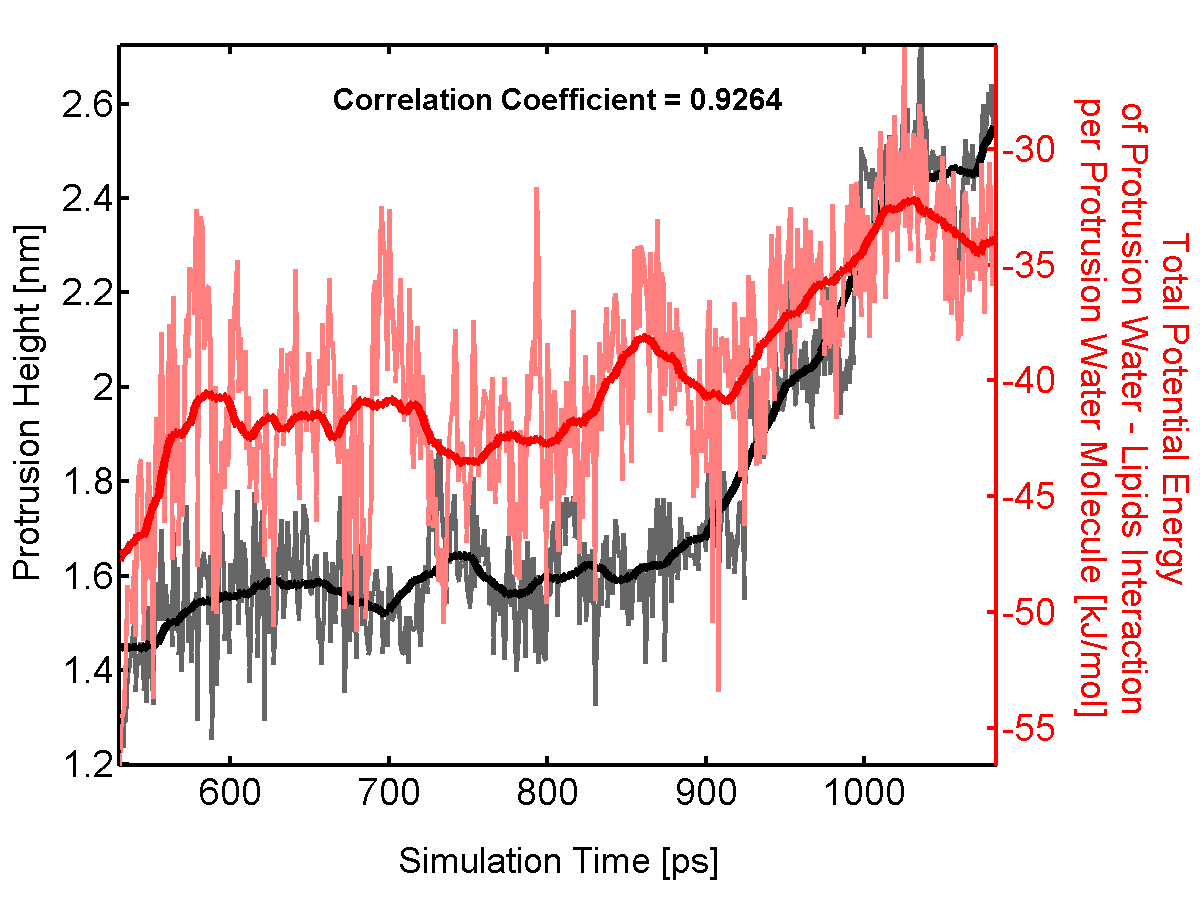
*
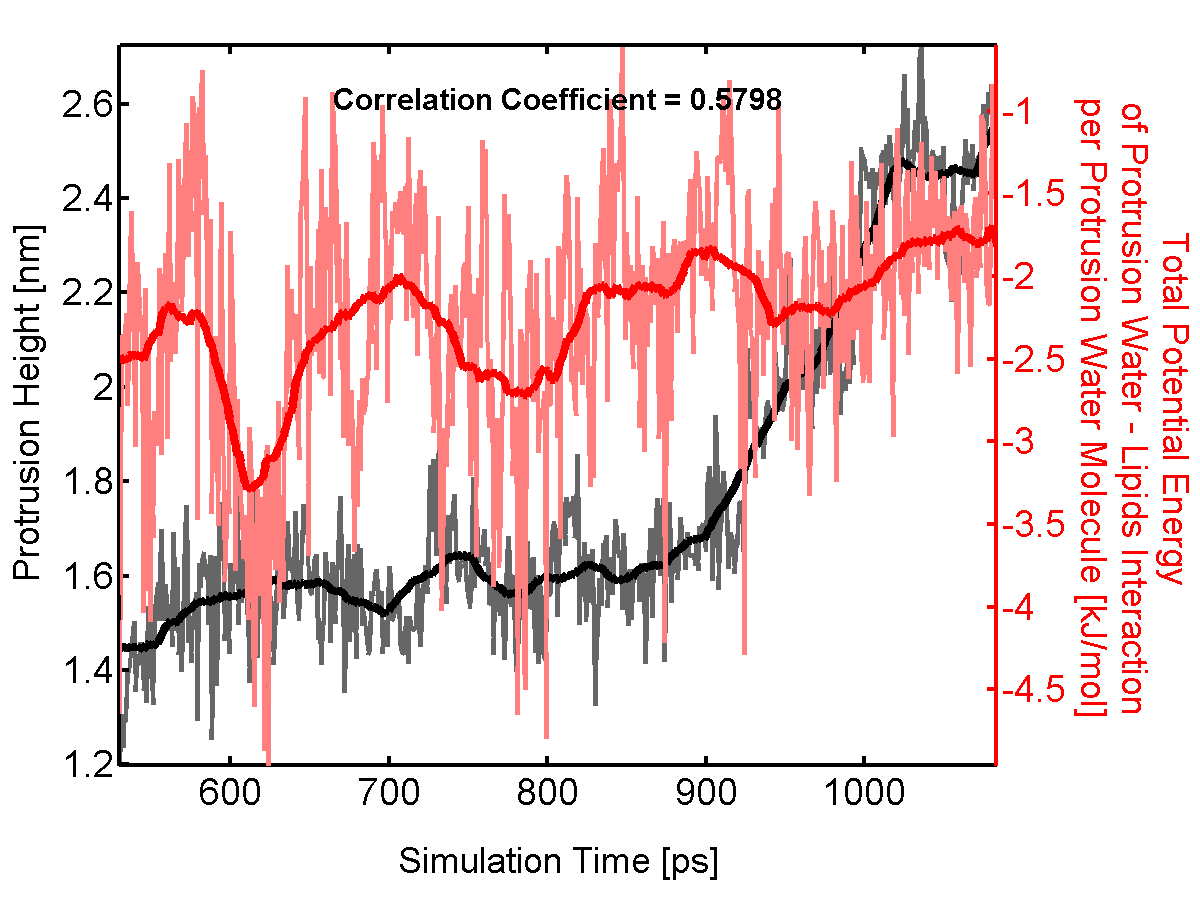
(a) (b)
