# Supplementary material for: Electric Field-Driven Water Dipoles: Nanoscale Architecture of Electroporation
Source: PLoS One. 2013 Apr 11;8(4):e61111. doi: 10.1371/journal.pone.0061111 (PMC3623848; doi:10.1371/journal.pone.0061111)
Supplement: Appendix S4 — Computing energetic interactions of planar and vertical configurations of seven dipoles. (DOCX) [file pone.0061111.s005.docx]

**SUPPORTING INFORMATION: Appendix S4**

**Title:** Electric Field-Driven Water Dipoles: Nanoscale Architecture of Electroporation

**Authors:** Mayya Tokman^†^, Jane HyoJin Lee^†^, Zachary A. Levine^‡^, Ming-Chak Ho^‡^, Michael E. Colvin^†^, P. Thomas Vernier^§^

*^†^School of Natural Sciences, University of California, Merced, CA, USA.*

*^‡^Department of Physics and Astronomy, University of Southern California, Los Angeles, CA, USA.*

*^§^Ming Hsieh Department of Electrical Engineering, University of Southern California,* *Los Angeles, CA, USA.*

**Corresponding Author:** Mayya Tokman, School of Natural Sciences, University of California, 5200 N. Lake Road, Merced, CA 95343, [mtokman@ucmerced.edu](mailto:mtokman@ucmerced.edu).

**APPENDIX S4: Computing energetic interactions of planar and vertical configurations of seven dipoles.**  In the example presented in the main text the planar and the vertical configurations of seven dipoles are compared (Fig. 4). The placement of the dipoles is chosen as follows. In both configurations we assume the spacing of dipoles to be consistent with the average spacing of SPC/E water model and set at 0.31 nm. The vertical configuration dipoles are all aligned with the external electric field, i.e. their dipole moment $\mu$ is taken to be parallel to $E$. For the planar dipole configuration the component of the dipole moment $\mu_{E}$ in the direction parallel to the electric field is assumed to be equal to the average dipole moment predicted by the Langevin-Debye formula

(S4)

$$<\mu_{E}>=\mu\left( \coth\left( \frac{\mu E}{kT} \right)-\frac{kT}{\mu E} \right),$$

which establishes the dependence of the dipole moment on the electric field. The angle of the planar component of the dipole moment $\mu_{P}=\mu-\mu_{E}$ with the planar coordinate axis is drawn from a uniform random distribution of values from 0 to $2\pi$.

The functional form of the total energy for both configurations includes two terms: the pairwise interaction between individual dipoles

$$U_{\mu_{1}\mu_{2}}=\frac{1}{4\pi\varepsilon_{0}}\frac{\left\| r_{12} \right\|^{2}\left( \mu_{1}\cdot\mu_{2} \right)-3\left( \mu_{1}\cdot r_{12} \right)\left( \mu_{2}\cdot r_{12} \right)}{\left\| r_{12} \right\|^{5}},$$

(S5)

where $\mu_{1}$ and $\mu_{2}$ are the dipole moment vectors and $r_{12}$ is the vector between the two dipoles, and the dipole-electric field interaction as defined by eqn. (S3) in Appendix S2. In addition, the vertical configuration of the dipoles also accounts for the desolvation energy required to remove seven dipoles from the bulk. Goncalves and Stassen [36] estimated the free energy of water solvation with molecular dynamics simulations. We use their result and estimate the free energy required to extract a water molecule from the bulk at -6.7 kcal/mol=-27.8 kJ/mol. Thus the total energy required to extract seven water dipoles from the bulk is approximately $U_{\mathrm{DS}}=-194.5$kJ/mol.
